# Concentration-Dependent Photoproduction of Singlet Oxygen by Common Photosensitizers

**DOI:** 10.3390/molecules30051130

**Published:** 2025-03-01

**Authors:** Grzegorz Szewczyk, Krystian Mokrzyński

**Affiliations:** Department of Biophysics, Faculty of Biophysics, Biochemistry and Biotechnology, Jagiellonian University in Krakow, 30-387 Krakow, Poland; krystian.mokrzynski@uj.edu.pl

**Keywords:** singlet oxygen, quantum yield, photosensitizers, methylene blue, TMPyP, perinaphthenone, ZnPc, Eosin Y, Rose Bengal, aggregation

## Abstract

Singlet oxygen quantum yield (Φ_Δ_) is a critical parameter in photodynamic studies, particularly for evaluating photosensitizers’ efficiency in diverse applications such as photodynamic therapy and environmental remediation. Standard photosensitizers, including Rose Bengal, Methylene Blue, and porphyrins, are widely employed as benchmarks for determining Φ_Δ_. However, accurate determination of Φ_Δ_ relies not only on the intrinsic properties of these photosensitizers but also on their experimental conditions, such as concentration. This study investigated the influence of photosensitizer concentration on singlet oxygen quantum yield using several standard photosensitizers. Our findings revealed a significant decrease in Φ_Δ_ with increasing photosensitizer concentrations across all tested compounds. This decline was attributed to self-quenching effects and molecular aggregation, which reduced the efficiency of energy transfer from the excited triplet state of the photosensitizer to molecular oxygen. The results emphasize the importance of optimizing photosensitizer concentration to ensure reliable Φ_Δ_ measurements and avoid underestimations. This work underscores the need to consider concentration-dependent effects in future studies to ensure accurate and reproducible outcomes.

## 1. Introduction

Singlet oxygen (^1^O_2_) plays a pivotal role in diverse applications, including photodynamic therapy and antimicrobial photoinactivation, where it is the main agent responsible for destroying or inactivating desired cells. The determination of singlet oxygen quantum yield (Φ_Δ_) is essential to evaluate the efficiency of photosensitizers used in these applications, such as photodynamic therapy or environmental photochemistry. It has been determined using several methods, both direct and indirect, both with advantages and limitations. In the indirect approach, external probes, either chemical or physical, are used. The former is based on trapping ^1^O_2_ with specific chemical acceptors that form products that can then be quantified using spectroscopic or chromatographic methods. Despite being widely used due to their low price and availability, this mode may be prone to different factors, including non-specific reactions of other reactive species with the probe [1]. The latter involves the determination of oxygen consumption. By monitoring the decrease in oxygen concentration during the photosensitized process, one may infer the production of singlet oxygen. On the other hand, this method requires precise measurement tools and may be affected by other oxygen-consuming processes occurring simultaneously [2]. Contrasting the indirect approach, the most reliable method for the quantification of singlet oxygen quantum yield is the time-resolved observation of its phosphorescence. Upon excitation, ^1^O_2_ emits weak phosphorescence in the near-infrared region, with a peak around 1270 nm. Using time-resolved spectroscopy, that emission can be detected and analyzed to provide accurate measurements of ^1^O_2_ photoproduction. The utmost advantage of that approach, besides time-resolved observations, is its specificity, which is due to its characteristic phosphorescence. Thus, interference from other reactive oxygen species and side reactions is minimized. Moreover, even if luminescence from other sources is present, it is relatively easy to differentiate its origin from the analysis of its lifetime. Due to characteristic peak absorbance, the presence of non-specific luminescence can be discriminated using a wavelength scan. Furthermore, time-resolved detection has better quantitative accuracy, allowing the precise determination of singlet oxygen lifetimes and yields and leading to more accurate quantum yield values. Real-time monitoring also enables the observation of ^1^O_2_ generation dynamics in the time-domain, providing insights into the kinetics of the photosensitization process.

With a direct approach, the determination of the exact quantum yield of the analyzed molecule requires the use of standard photosensitizers with a known efficacy of singlet oxygen photogeneration in a given environment. Their efficacy is determined by multiple factors, including photophysical properties and environmental conditions. Most photosensitizers have a cyclic compound that allows absorption in the visible region of light but usually decreases the hydrophilicity of the molecule. Photosensitizers are known to aggregate in water environments [3,4,5]. The aggregation of photosensitizers is a critical phenomenon that negatively affects their ability to generate singlet oxygen. In both polar and non-polar solvents, aggregation can reduce quantum yield due to quenching mechanisms such as π-π stacking and intramolecular charge transfer. For example, studies have shown that disaggregated photosensitizer monomers are significantly more effective in producing singlet oxygen than their aggregated counterparts [6]. This phenomenon holds substantial implications for photodynamic therapy. Aggregation can impede photosensitizer performance, leading to reduced therapeutic efficacy. Research on phenothiazine derivatives demonstrated that monomeric forms exhibited higher singlet oxygen yields, emphasizing the importance of stabilization strategies to prevent aggregation [7]. Similarly, studies on DNA-binding photosensitizers highlighted a decrease in singlet oxygen yield due to steric hindrance in aggregated states [8]. Aggregation-induced emission photosensitizers represent a promising solution to this challenge. These systems generate singlet oxygen efficiently in their aggregated states, circumventing typical quenching effects observed with conventional photosensitizers [9]. However, precise molecular engineering is required to balance aggregation-induced quenching and emission properties, particularly in varying solvent environments [10].

In this study, the role of photosensitizer concentration and possible aggregation on the photogeneration of singlet oxygen was assessed using methylene blue (MB), Perinaphthenone (PN), 5,10,15,20-Tetrakis(1-ethyl-4-pyridinio)porphyrin-tetra(p-toluenesulfonate) (TMPyP), zinc phthalocyanine (ZnPc), and Eosin Y. The aim of this study was to identify possible issues that may occur as part of the evaluation of singlet oxygen photogeneration. In the study, photogeneration of singlet oxygen was observed using time-resolved detection of the characteristic phosphorescence of singlet oxygen at 1270 nm, with quantum yields determined using a comparative method. The findings of this work might contribute to the re-evaluation of quantum efficiency values from a wide range of photosensitizing molecules, which, depending on the concentration of the employed standard, could have been overestimated or underestimated. These findings may also provide valuable insights into optimizing photosensitizer formulations for enhanced performance in photodynamic therapy.

## 2. Results

**Figure 1:**
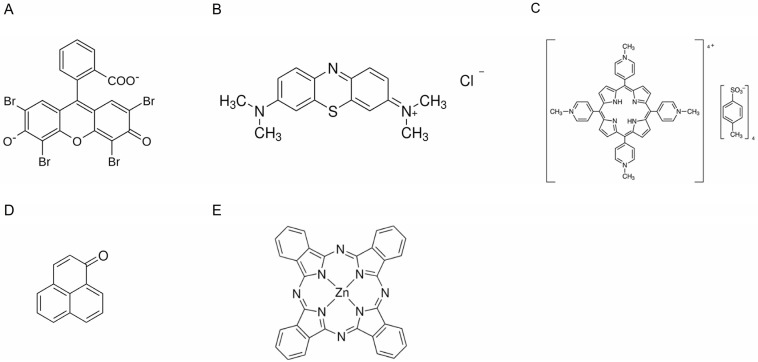
Chemical structures of employed photosensitizers: Eosin Y (**A**), Methylene Blue (**B**), TMPyP (**C**), PN (**D**), ZnPc (**E**).

A range of the most used photosensitizing standards were employed to demonstrate the effect of PS concentration on their ability to photogenerate singlet oxygen. Previously, Rose Bengal was found to exhibit non-linear dependence between the detected signal of singlet oxygen and the concentration of the molecule. This non-linear behavior occurred even at the concentrations found in the literature employed as a standard during the quantum yield determination of other molecules [5]. Here, these effects were analyzed in D_2_O-based solutions of MB, Eosin Y, and TMPyP, as well as THF-based solutions of PN and ZnPc (Figure 1).

### 2.1. Optical Properties

**Figure 2:**
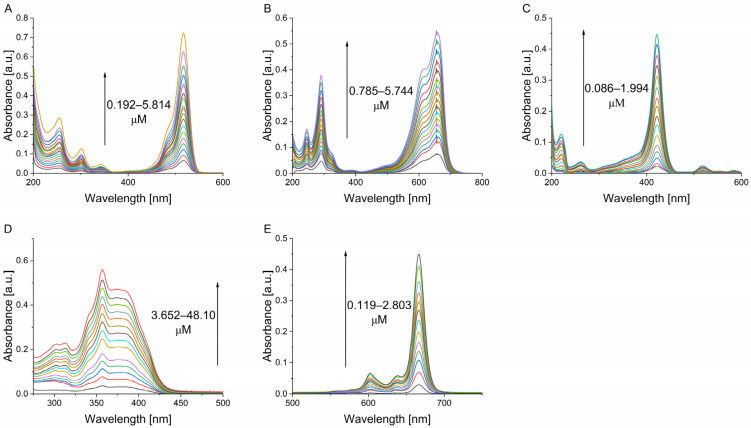
Optical properties of analyzed photosensitizers: Eosin Y (**A**), Methylene Blue (**B**), TMPyP (**C**) in PBS-D_2_O and PN (**D**), ZnPc (**E**) in THF.

There are no ubiquitous photosensitizing standards that absorb light in the whole UVA-VIS/near-IR spectrum. Thus, a range of molecules, each with specific optical parameters, covers the spectrum (Figure 2). PN exhibits its maximum absorbance in the UVA region (356 nm in THF). TMPyP demonstrates typical porphyrin absorbance peaks at the Soret band (around 422 nm in D_2_O-PBS solutions). Eosin Y absorbs light most efficiently in the green part of visible light (516 nm in D_2_O-PBS solutions), while MB and ZnPC show their peak absorbance in the red part of the spectrum (660 nm in D_2_O-PBS solutions and 666 nm in THF-based solutions, respectively). In this work, the concentrations of the solutions of the examined photosensitizers were calculated based on their absorbance peak values. Using the Lambert–Beer law and knowing the extinction coefficients for each compound, the exact concentration of each solution was determined prior to the determination of singlet oxygen photogeneration. According to the Lambert–Beer law, concentration and absorbance dependence exhibit linear behavior in the range of 0.1–1. Lower values might be affected by hardware-related noise, while at higher absorbance values, the concentration dependence tends to stray from a linear relationship.

In order to minimize the potential error resulting from the abovementioned confinements, only samples where the absorbance values in the peak value were in the range of 0.05–0.6 were used in further analysis (Figure 2).

The presence of dimeric or higher order aggregates is often accompanied by either absorption shift to the lower wavelengths (hypsochromic effect) or higher wavelengths (bathochromic effect) or broadening of absorption spectra, depending on the aggregation type. In the range of concentrations used in this study, the measured absorbance values increased linearly with the amount of titrated solutions of photosensitizers (Figure 2). Moreover, none of the abovementioned effects was observed in either Eosin Y (Figure 2A) and TMPyP (Figure 2C) in PBS-D_2_O or PN (Figure 2D) and ZnPc (Figure 2E) in THF. As expected, some potential aggregation was observed for PBS-D_2_O-based MB solutions, where a decrease in the monomer-to-dimer ratio (660 nm/615 nm) was observed (1.63 to 1.37) which is consistent with previously reported data [11].

### 2.2. Singlet Oxygen Phosphorescence vs. Concentration of Photosensitizer

**Figure 3:**
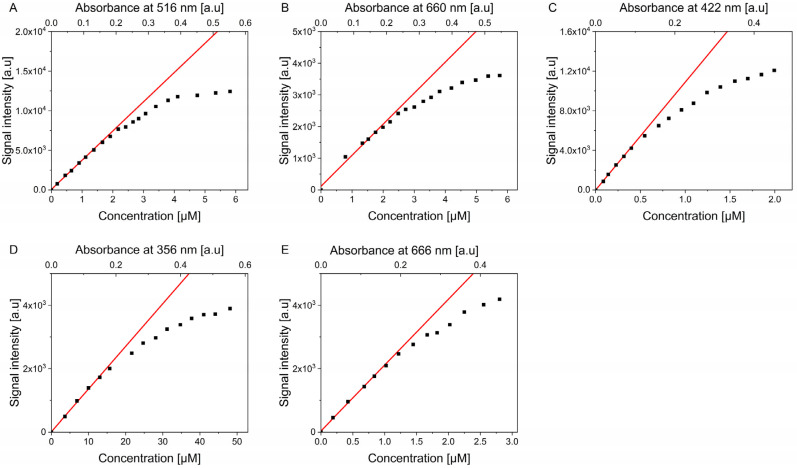
Intensity of singlet oxygen phosphorescence with increasing concentrations of photosensitizers: Eosin Y in PBS-D_2_O (**A**); Methylene Blue in PBS-D_2_O (**B**); TMPyP in PBS-D_2_O (**C**); PN in THF (**D**); ZnPc in THF (**E**). The red line denotes the linear fit to the linearly aligned points at lower concentrations. The experiments were performed in triplicate (n = 3) yielding similar results; however, to increase clarity, only a single series for each photosensitizer is plotted.

The relation between the intensity of the singlet oxygen signal obtained using time-resolved phosphorescence detection was plotted against the concentration of the photosensitizer (Figure 3). Eosin Y (Figure 3A) and MB (Figure 3B) in PBS-D_2_O exhibited linear dependence up to approximately 2 μM concentration, with the first signs of deviating from linearity observed at higher concentrations. Similar behavior was also observed in D_2_O-based solutions, where the linear range for both photosensitizers spanned approximately 2 μM (Supplementary Figure S1). TMPyP was found to lose its linear response at a significantly lower concentration of approximately 0.5 μM with a non-linear increase in signal intensity observed afterward. 

Rather surprisingly, similar non-linear behavior was observed for PN (Figure 3D) and ZnPc (Figure 3E) in THF. The former tends to deviate from linear dependence at much higher concentrations compared to previously mentioned photosensitizers; however, it is important to note that its molar extinction coefficient is an order of magnitude lower than those compounds, thus the deviation starts at peak absorbance at just 0.2. The latter was found to be non-linearly dependent with concentrations higher than approximately 1.2 μM. It should be noted that the concentrations were calculated based on absorbance values and molar extinction coefficients.

### 2.3. Singlet Oxygen Quantum Yield vs. The Concentration of Photosensitizer

Due to their wide absorbance spectrum, these standards of singlet oxygen can be employed to determine the quantum yields of another molecule of interest using the comparative method. The comparative method assumes that the same amount of photons are absorbed by both the examined and standard molecules, making it necessary to provide the same absorbance value for both. However, when performing such experiments, the excitation wavelength is tailored to the examined molecule’s peak absorbance and not the standard; thus, the standard is often excited far from its absorbance peak. This action may result in using higher concentrations of a standard photosensitizer, which is potentially beyond the linear range of singlet oxygen intensity vs. concentration dependence (Figure 3).

**Figure 4:**
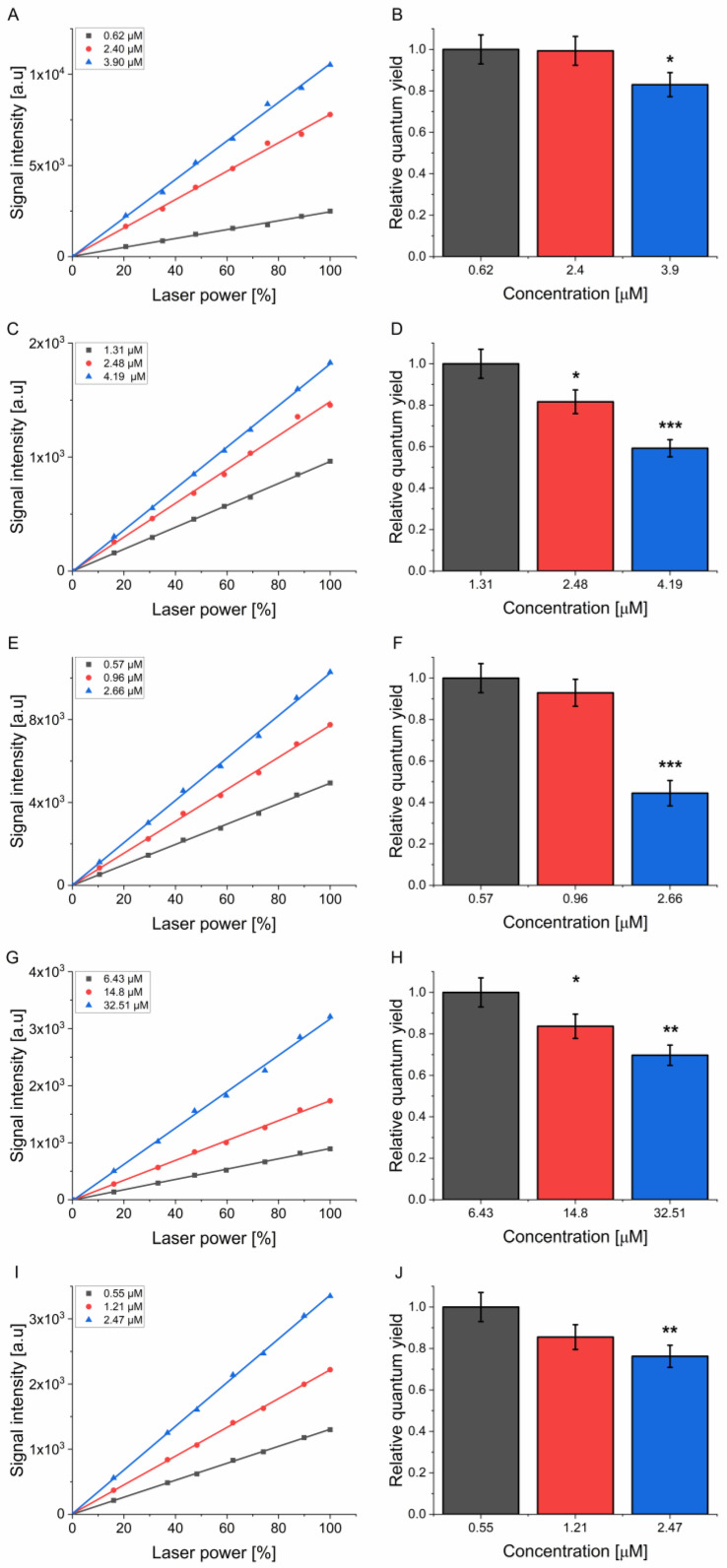
Singlet oxygen quantum yields determined at different photosensitizer concentrations (left column). Calculated ratios (normalized to lowest concentration) of quantum yield at given concentrations of PS (right column). (**A**,**B**)—Eosin Y (in PBS-D_2_O); (**C**,**D**)—MB (in PBS-D_2_O); (**E**,**F**)—TMPyP (in PBS-D_2_O); (**G**,**H**)—PN (in THF); (**I**,**J**)—ZnPC (in THF). The experiments were performed in triplicate (n = 3), yielding similar results; however, to increase clarity, only a single series for each photosensitizer is plotted in **A**,**C**,**E**,**G**,**I**. Statistical significance was tested using ANOVA with Tukey’s post hoc test, with asterisks denoted as follows: * *p* < 0.05, ** *p* < 0.01, *** *p* < 0.001.

In an ideal situation, the intensity of singlet oxygen phosphorescence should be linearly aligned with the concentration of excited molecules. Based on the results demonstrated in Figure 3, the quantum yield of photosensitizers was assessed to determine whether the relative quantum yield (quantum yield normalized to the sample concentration) remains the same regardless of increasing concentrations (Figure 4, Supplementary Figure S2). The concentrations used in quantum yield determination experiments were selected to represent different behaviors as observed in Figure 3. The lowest concentration represented the linear region of dependence, the medium concentration represented the region where the weakest non-linearity was observed, and the highest concentration represented the strongly non-linear region. All of the examined photosensitizers demonstrated a lower singlet oxygen signal than might have been expected if the relationship with concentration was indeed linear. That behavior resulted in quantum yields being lower with increasing concentrations than the expected value (Table 1).

A xanthene dye Eosin Y was found to exhibit a 17% drop in the yield of singlet oxygen photoproduction compared to the lowest concentration used when samples were examined in PBS-D_2_O. The effect was even more pronounced when D_2_O was used as a solvent, with a more than two-fold higher drop in the quantum yield of singlet oxygen photoproduction at a comparable concentration difference. Time-resolved laser flash photolysis performed for Eosin Y showed a strong reduction in the lifetime of the excited triplet state with its increasing concentration (Supplementary Figure S3). On the other hand, the presence of phosphate-buffered saline in the solvent was not crucial in the case of Methylene Blue, where a similar drop in quantum yield was observed. It is worth noting that at the highest concentration used here, the calculated yield of singlet oxygen photoproduction was more than 40% lower than the expected one. Another xanthene dye previously studied by our group, Rose Bengal, exhibited similar limiting effects at increasing concentrations on singlet oxygen quantum yield [5]. TMPyP exhibited the most dramatic decrease in singlet oxygen photogeneration yield among all compounds studied in PBS-D_2_O, with a more than 50% decrease between the highest and lowest concentrations. An explanation for such a dramatic decrease observed for TMPyP is its previously reported tendency to stick to laboratory glass, which affects its concentration in solution [12]. The trend observed earlier for THF-based PN and ZnPc samples (Figure 3D,E) was confirmed during quantum yield determinations (Figure 4, Table 1). Both compounds showed reduced values of quantum yields beyond the linear region of singlet oxygen intensity vs. concentration (Figure 3D,E) with a 30% difference detected between the highest and lowest concentrations used.

**Table 1:** Differences between expected and calculated values of the quantum yield of examined photosensitizers.

Photosensitizer	Solvent	Concentration [μM]	Expected Quantum Yield	Calculated Quantum Yield	Difference [%]
Eosin Y	D_2_O-PBS	0.62	0.50 [13]	0.50 *	-
		2.40		0.497	0.60
		3.90		0.415	17.00
	D_2_O	0.72		0.50 *	-
		2.02		0.415	17.00
		4.15		0.323	35.40
Methylene Blue	D_2_O-PBS	1.31	0.52 [13,14]	0.52 *	-
		2.48		0.425	18.27
		4.19		0.308	40.77
	D_2_O	0.63		0.52 *	-
		1.49		0.509	2.12
		2.74		0.419	19.42
Rose Bengal ^1^	D_2_O-PBS	0.87 ^1^	0.76 [13,14]	0.76 ^1^	-
		1.70 ^1^		0.684 ^1^	10.00
		3.39 ^1^		0.586 ^1^	22.89
TMPyP	D_2_O-PBS	0.57	0.77 [15]	0.77 *	-
		0.96		0.716	7.01
		2.66		0.343	55.45
Perinaphthenone	THF	6.43	0.98 ^2^ [16]	0.98 *	-
		14.8		0.820	16.33
		32.51		0.683	30.31
ZnPc	THF	0.55	0.53 [17]	0.53 *	-
		1.21		0.500	5.66
		2.47		0.370	30.19

^1^ Data previously published by the authors [5]. ^2^ Based on data for AcN solutions [16]. * Based on literature data.

## 3. Discussion

Searching for new compounds that might be utilized for their photosensitizing properties is a never-ending quest. Singlet oxygen is a key reactive species mediating photodynamic action; thus, the quantum yield of its photoproduction is usually considered an important parameter for photosensitizers from the therapeutic perspective.

Due to the method’s nature, direct detection of characteristic phosphorescence of the singlet oxygen at 1270 nm is the most accurate method of singlet oxygen quantum yield determination. By plotting the intensity of singlet oxygen signal against laser power for the analyzed molecule and a standard photosensitizing agent, one can precisely quantify the quantum yield, provided that the experiments are carried out under similar conditions (using the same solvent, adjusting the absorbance at the analyzed wavelength to the same value, and exciting with the same pulse of light). The scientific community cannot underestimate magnificent works such as those by Wilkinson [14] and Redmond and Gamlin [13] for collecting and delivering data on the significant number of photosensitizers that might be used as reference standards for singlet oxygen determination. Unfortunately, it is rather a privilege, and not a standard situation, that one might excite the reference standard at its peak absorbance when determining the quantum yield of another molecule. Due to the different optical properties of analyzed molecules and standards, only a partial overlap of absorbance usually occurs.

The results of the current study suggest that one must be especially careful when determining quantum yields of singlet oxygen photogeneration, as the process might be prone to more factors than previously expected. All molecules analyzed in this work are commonly used as reference standards for singlet oxygen photoproduction (Figure 1). All of these photosensitizing molecules demonstrated a reduction in the quantum yield of singlet oxygen photoproduction with increasing concentration (Figure 3 and Figure 4, Table 1). This behavior indicates that when used as a reference molecule, these compounds must be used at the lowest possible concentration when the signal of singlet oxygen photoproduction aligns linearly with the concentration of the photosensitizer. That being said, when these photosensitizing standards are used beyond the linear region, the determined quantum yield of singlet oxygen photoproduction for another molecule may be seriously overestimated or underestimated.

Although similar observations were made previously, to our knowledge, they were not quantitively described apart from work on RB, which was recently published by our group [5]. A probable explanation for the observed reduction in singlet oxygen photoproduction is the increased rate of triplet excited state self-quenching with the increasing concentration of the molecule. That mechanism was previously shown for Rose Bengal, where the reduced lifetimes of the transient triplet states were observed with the increasing concentrations of the photosensitizer not only in solution but also bound to serum albumin [18]. Another plausible reasoning could be related to the formation of transient charge-transfer complexes, as previously suggested for TMPyP [12], which would change the ratio of the excited triplet state deactivated by energy transfer to molecular oxygen. However, the reduction rate of photoproducing singlet oxygen was rather surprising. Other mechanisms potentially involved in the observed phenomena include the formation of dimeric and/or higher-order aggregates by photosensitizers. All the compounds examined in this study were previously described as being prone to aggregation accompanied by hypochromic and bathochromic shifts in absorbance, reduced absorbance at the peak, or broadening of peaks [11,19,20]. Dimerization would reduce the distribution of photosensitizer molecules in the solvent. It could also increase the autoquenching of molecules, as previously demonstrated for Rose Bengal and Methylene Blue [11]. Indeed, changes in absorbance suggesting a drop in monomer-to-dimer ratios were observed in this study for MB (Figure 2B) and previously reported for RB [5]. However, no signs of aggregation in optical shifts were observed for TMPyP, PN, or ZnPc (Figure 2). On the other hand, it must be stressed that increasing the concentration of photosensitizers can affect the yield of energy transfer from the excited triplet state to molecular oxygen.

Considering the data obtained in this study, especially the observations shown in Figure 3, it is recommended to keep the concentrations of photosensitizers in a linear range for concentration-dependent singlet oxygen photogeneration. In the case of photosensitizers used in this study, the linear region stretches up to the point where the peak absorbance of respective PS does not exceed approximately 0.2. One must be aware that in real-life applications, the concentration of the standard is adjusted to match the peak absorbance of the examined molecule. However, maintaining the standard’s concentration within the linear range may not be possible. That kind of determination could still provide useful information about the singlet oxygen quantum yield but measurement errors may increase.

The data presented in this work emphasize the need to consider the concentration of photosensitizing reference standards, which, by several possible mechanisms, influence the validity of quantum yields determined for other molecules. The findings may prompt the need to reconsider and reexamine quantum yields of singlet oxygen photogeneration for several molecules for which the currently applied values might be overestimated or underestimated.

## 4. Materials and Methods

### 4.1. Materials

The following materials were purchased from Merck (Steinheim, Germany): Methylene Blue (MB), Phenalene-1-one (PN), meso-Tetra(N-methyl-4-pyridyl)porphyrin (TMPyP), Zinc Phthalocyanine (ZnPC), Eosin Y, deuterium oxide (D_2_O), and Tetrahydrofuran (THF). Phosphate-buffered saline D_2_O (PBS-D_2_O) was prepared by adding 127 mM NaCl, 2.7 mM KCL, 8 mM Na_2_HPO_4_, and 2 mM KH_2_PO_4_ (all purchased from POCH, Gliwice, Poland) to D_2_O.

### 4.2. Sample Preparation

Small portions of 1 mM photosensitizer solutions prepared in D_2_O, PBS-D_2_O, or THF were titrated to 1.5 mL of the respective solvent. The exact concentrations of photosensitizers in the examined samples were calculated based on the absorbance measured with HP 8453 UV–Vis spectrophotometer (Hewlett-Packard, Palo Alto, CA, USA) using molar extinction coefficients for MB (ε = 9.69 × 10^4^ M^−1^cm^−1^), PN (ε = 1.15 × 10^4^ M^−1^cm^−1^), TMPyP (ε = 2.2 × 10^5^ M^−1^cm^−1^), ZnPC (1.6 × 10^5^ M^−1^s^−1^) and EosinY (ε = 9.5 × 10^4^ M^−1^s^−1^).

### 4.3. Direct Time-Resolved Singlet Oxygen Phosphorescence

The solvents were placed in quartz cuvettes and titrated with small portions of photosensitizers. Samples were constantly stirred using a magnetic stirrer and excited for 30 s with 516 nm (Eosin Y), 660 nm (Methylene Blue), 422 nm (TMPyP), 356 nm (PN), or 666 nm (ZnPc) laser pulses generated by an integrated nanosecond DSS Nd:YAG laser system equipped with a narrow-bandwidth optical parameter oscillator (NT242-1k-SH; Ekspla Vilnius, Lithuania) operating at 1 kHz repetition rate. The excitation beam was attenuated with five layers of wire mesh (~35% reduction each) to reduce the power of the excitation beam. Photoluminescence detection and analysis were performed using a setup described elsewhere [21]. The initial intensity of singlet oxygen phosphorescence was determined using a formula described earlier [5]. Quantum yields were determined by performing measurements with a laser beam additionally attenuated using a series of filters with increasing transmittance to obtain a regression line. Three independent measurements were performed for all examined photosensitizers. The statistical significance between groups was determined using ANOVA with Tukey’s post hoc test employing OriginPro 2024b software. Differences were considered statistically significant at *p* < 0.05.

### 4.4. Laser-Flash Photolysis

The measurements were conducted using the custom-built setup described earlier [22]. In brief, a phosphate-buffered (pH 7.2) D_2_O solution of Eosin Y in a 1 cm optical path quartz fluorescence cuvette (QA-1000; Hellma, Mullheim, Germany), was excited with 516 nm pulses generated by an integrated nanosecond DSS Nd:YAG laser system equipped with a narrow bandwidth optical parametric oscillator (NT342; Ekspla, Vilnius, Lithuania). Absorbance changes were monitored perpendicularly to the excitation using a PMT module (R928, Hamamatsu, Japan) equipped with a monochromator to provide wavelength selectivity.

## Data Availability

The data is available from the corresponding author on request.

## Supplementary Materials

The following supporting information can be downloaded at: https://www.mdpi.com/article/10.3390/molecules30051130/s1, Figure S1: Intensity of singlet oxygen phosphorescence at increasing concentrations of photosensitizers: Eosin Y in D_2_O (A), Methylene Blue in D_2_O (B). The red line denotes the linear fit to the linearly aligned points at lower concentrations; Figure S2: Singlet oxygen quantum yields determined for different concentrations of photosensitizer (left column). Calculated ratios (normalized to lowest concentration) of quantum yield at given concentrations of PS (right column). A,B—Eosin Y in D_2_O, C,D—MB in D_2_O. The experiments were performed in triplicate (n = 3), yielding similar results; however, to increase clarity, only a single series for each photosensitizer is plotted in A,C,E,G,I. Statistical significance was tested using ANOVA with Tukey’s post hoc test, and asterisks are denoted as follows: * *p* < 0.05, *** *p* < 0.001. Figure S3: Changes in the transient absorption of Eosin Y at 580 nm determined using time-resolved laser flash photolysis (A). Decay times of triplet excited states (B). Statistical significance was tested using ANOVA with Tukey’s post hoc test, and asterisks are denoted as follows: *** *p* < 0.001.

## Abbreviations

The following abbreviations are used in this manuscript:

D2O	Deuterium oxide, heavy water
MB	Methylene Blue
PBS-D2O	Phosphate buffered saline-deuterium oxide
PN	Perinaphthenone
RB	Rose Bengal
THF	Tetrahydrofuran
TMPyP	5,10,15,20-Tetrakis(1-methyl-4-pyridinio)porphyrin-tetra(p-toluenesulfonate)
UV–Vis	Ultraviolet–visible range of electromagnetic radiation
ZnPc	Zinc phthalocyanine
