# Supplementary material for: Concentration-Dependent Photoproduction of Singlet Oxygen by Common Photosensitizers
Source: Molecules. 2025 Mar 1;30(5):1130. doi: 10.3390/molecules30051130 (PMC11901654; doi:10.3390/molecules30051130)
Supplement: Supplementary file 1 [file molecules-30-01130-s001.zip › molecules-3481528-supplementary.pdf]

Supplementary data
for

Concentration-dependent photoproduction of singlet oxygen by common photosensitizers

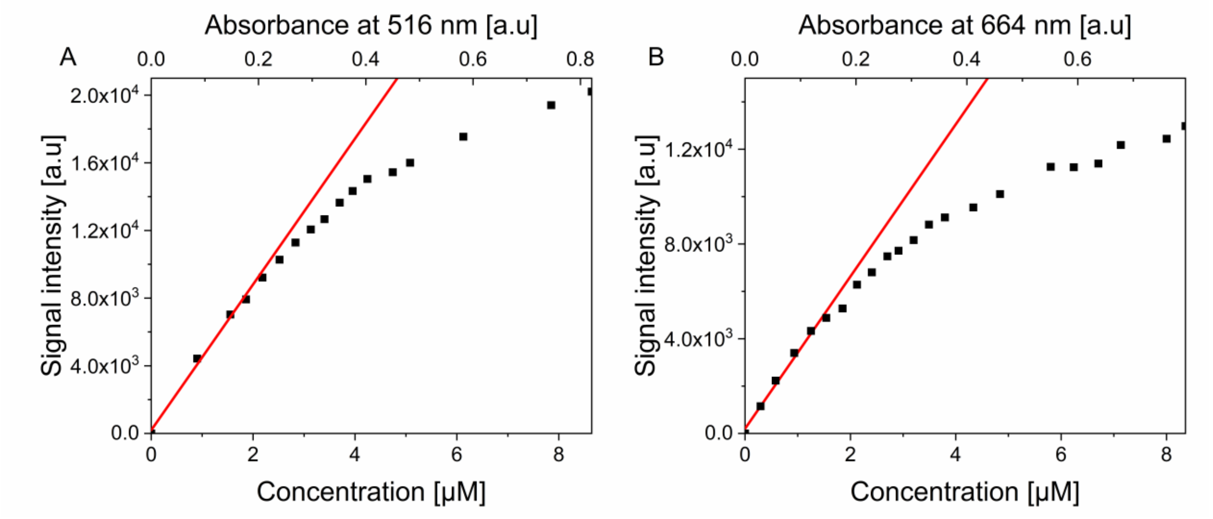

Supplementary Figure S1. Intensity of singlet oxygen phosphorescence with the increasing concentrations of photosensitizers: Eosin Y in D_2O (A), Methylene Blue in D_2O (B). Red line denotes the linear fit to the linearly aligned points at lower concentrations.

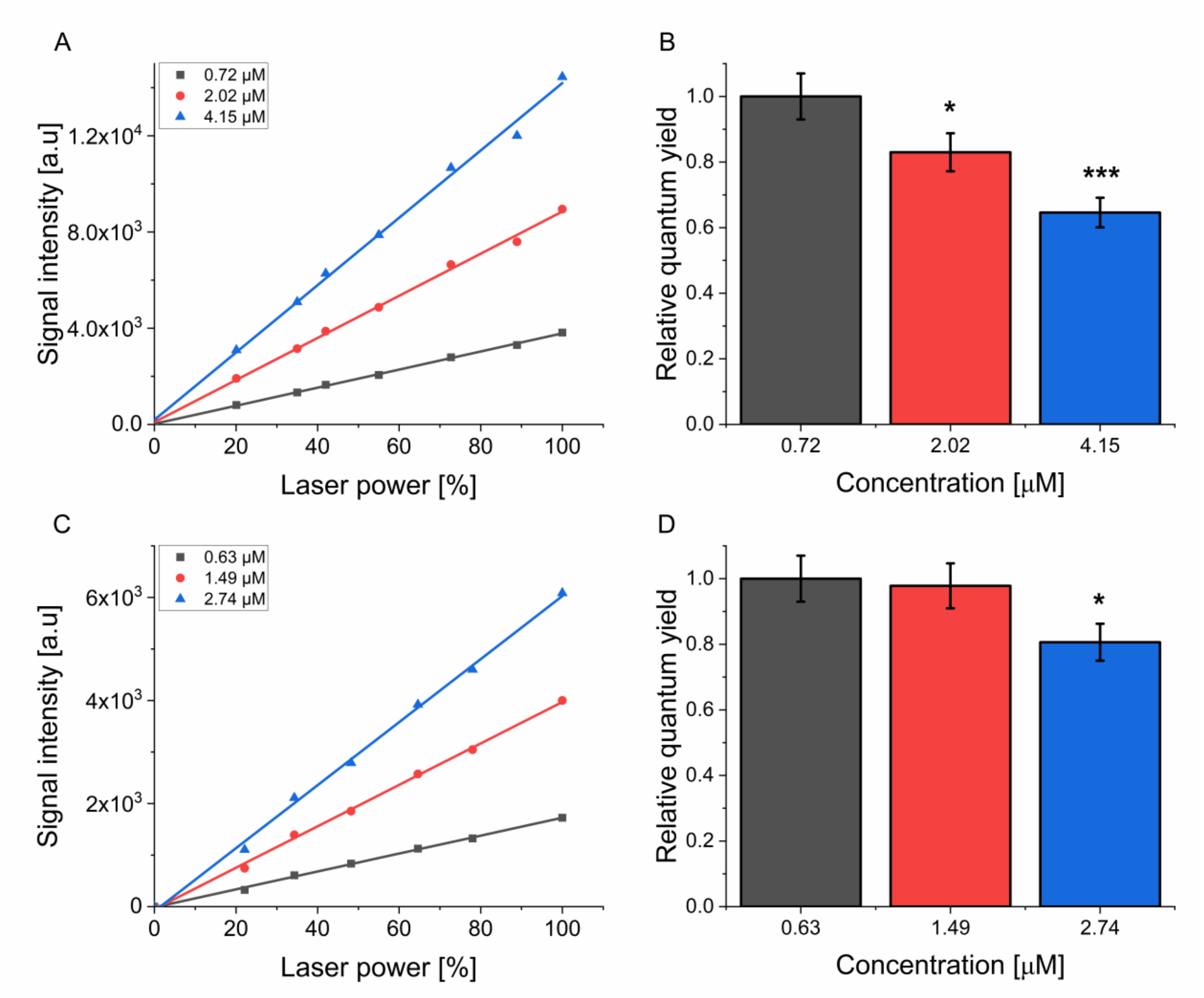

Supplementary Figure S2. Singlet oxygen quantum yields determined for different concentrations of photosensitizer (left column). Calculated ratios (normalized to lowest concentration) of quantum yield at given concentration of PS (right column). A,B – Eosin Y in D_2O, C,D – MB in D_2O. The experiments were performed in triplicates (n=3) yielding similar results, however, to increase clarity only a single series for each photosensitizer is plotted in A,C,E,G,I. Statistical significance was tested using ANOVA with Tukey's *post-hoc* test and asterisks denote as follows: * $p<0.05$, ** $p<0.01$, *** $p<0.001$

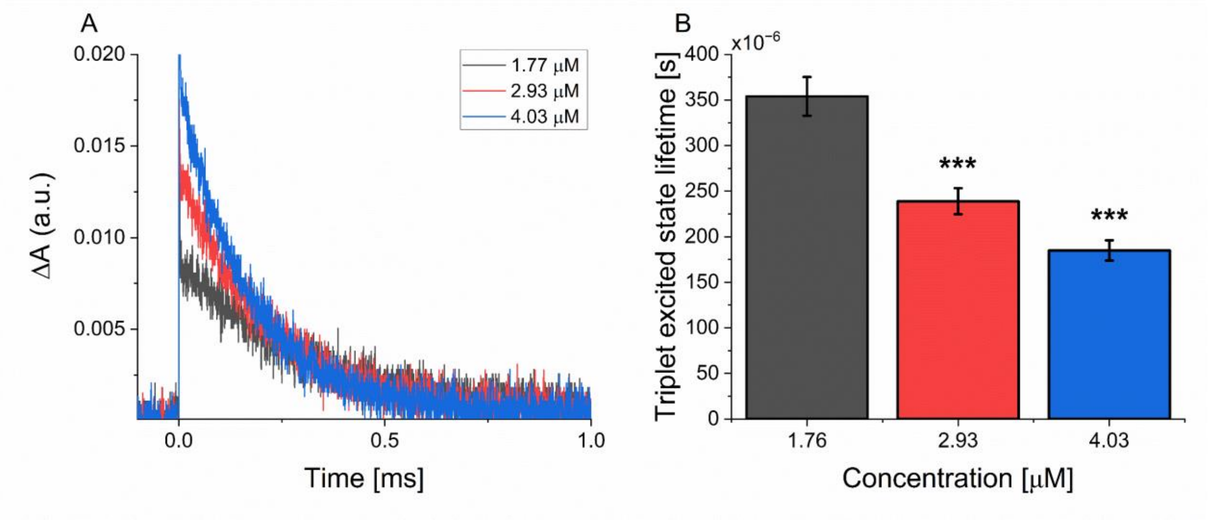

Figure S3: Changes in the transient absorption of Eosin Y at 580 nm determined using time-resolved laser flash photolysis (A). Decay times of triplet excited states (B). Statistical significance was tested using ANOVA with Tukey's *post-hoc* test and asterisks denote as follows: *** $p<0.001$
